# Beirut Ammonium Nitrate Blast: Analysis, Review, and Recommendations

**DOI:** 10.3389/fpubh.2021.657996

**Published:** 2021-06-04

**Authors:** Samar Al-Hajj, Hassan R. Dhaini, Stefania Mondello, Haytham Kaafarani, Firas Kobeissy, Ralph G. DePalma

**Affiliations:** ^1^Health Management and Policy, Faculty of Health Sciences, American University of Beirut, Beirut, Lebanon; ^2^Department of Environmental Health, Faculty of Health Sciences, American University of Beirut, Beirut, Lebanon; ^3^Department of Biomedical and Dental Sciences and Morphofunctional Imaging, University of Messina, Messina, Italy; ^4^Division of Trauma, Emergency Surgery and Surgical Critical Care. Massachusetts General Hospital, Boston, MA, United States; ^5^Department of Biochemistry & Molecular Genetics, Faculty of Medicine, American University of Beirut, Beirut, Lebanon; ^6^Office of Research and Development, Department of Veterans Affairs, Washington, DC, United States

**Keywords:** ammonium nitrate explosion, blast injury, Beirut, emergency preparedness, health hazard, traumatic brain injury

## Abstract

A massive chemical detonation occurred on August 4, 2020 in the Port of Beirut, Lebanon. An uncontrolled fire in an adjacent warehouse ignited ~2,750 tons of Ammonium Nitrate (AN), producing one of the most devastating blasts in recent history. The blast supersonic pressure and heat wave claimed the lives of 220 people and injured more than 6,500 instantaneously, with severe damage to the nearby dense residential and commercial areas. This review represents one of the in-depth reports to provide a detailed analysis of the Beirut blast and its health and environmental implications. It further reviews prior AN incidents and suggests actionable recommendations and strategies to optimize chemical safety measures, improve emergency preparedness, and mitigate the delayed clinical effects of blast and toxic gas exposures. These recommended actionable steps offer a starting point for government officials and policymakers to build frameworks, adopt regulations, and implement chemical safety protocols to ensure safe storage of hazardous materials as well as reorganizing healthcare system disaster preparedness to improve emergency preparedness in response to similar large-scale disasters and promote population safety. Future clinical efforts should involve detailed assessment of physical injuries sustained by blast victims, with systemic mitigation and possible treatment of late blast effects involving individuals, communities and the region at large.

## Introduction

Chemical explosions cause large disasters and civilian mass casualties. Throughout history, chemical explosions, particularly those caused by Ammonium Nitrate (AN) have caused tragedies with devastating human and infrastructure loss, disturbing all functional aspects of affected communities (1, 2). The latest AN blast in Beirut was categorized as the third most devastating urban explosions of all time after the Hiroshima and Nagasaki nuclear bombings at the end of world war II (3).

The Beirut explosion created a massive blast that produced a 140 m wide crater and an earthquake of a 3.3 magnitude on a Richter scale, killing nearly 220 individuals and injuring more than 6,500 instantly, while leaving ~300,000 people homeless (4). Over and above the human tragedy, this large disaster damaged 9 of the capital's hospitals (5) and hampered access to healthcare for nearly 160,000 patients. The blast further damaged schools, commercial centers, museums, news organizations, and foreign embassies which hindered communication and exchange of essential information with local staff, residents and travelers with pressing need for accurate and timely instructions on urgent matters. The blast estimated economic burden exceeds 6.7 Billion US dollars (6). In addition, the blast AN ignition released toxic gases that posed a real threat to Beirut's 2.4 million residents, particularly when mixed with sea humidity and dust particles emitted from the demolished and collapsed buildings.

This review complements recent reports (7–18) and further provide an in-depth analysis of the Beirut blast and its health and environmental implications, and suggests actionable recommendations and strategies.

## Beirut Blast: Health And Environment Implications

### Beirut Blast: Context and Timeline

**Figure 1:**
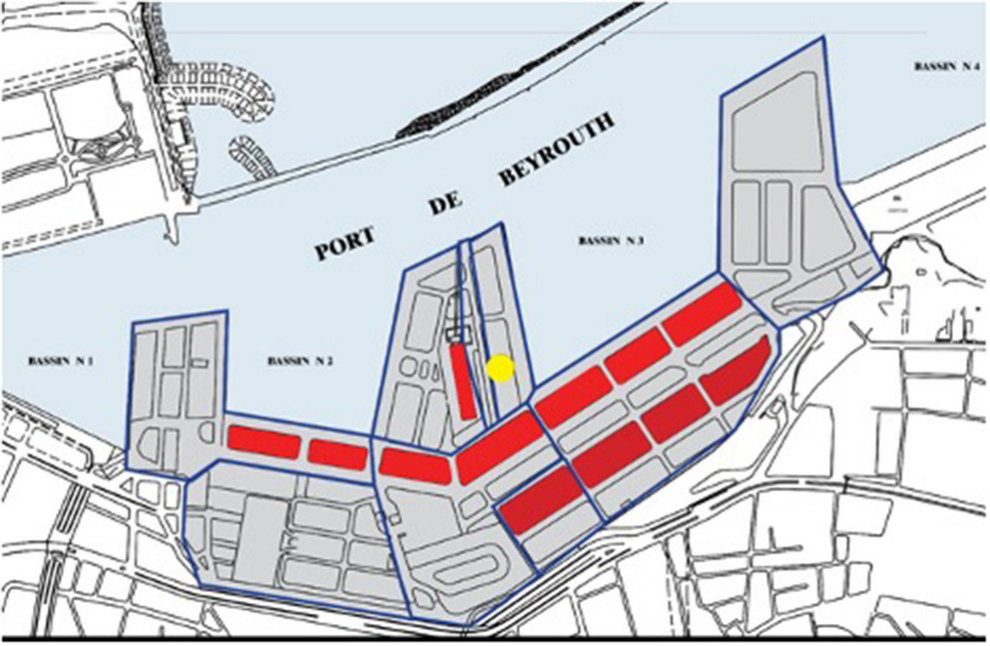
Blast location at the port of Beirut and adjacent warehouses. The yellow circle refers to the blast location in hangar 12 next to the grain silos and fireworks warehouses. Red rectangles depict the warehouses.

Lebanon, an upper middle-income country, is located on the Mediterranean Sea. The port of Beirut is Lebanon's main entry point in its capital city Beirut with its strategic geographic location at the nexus of three continents: Europe, East Asia, and Africa. The port is one of the largest ports in the Middle East, a regional hub providing major imports for the Eastern Mediterranean region including Syria, Jordan, Iraq, and the Persian Gulf States. It spreads over a 1.2 Km^2^ area with terminals for passengers, general cargo, and containers, in addition to a grain silo and a duty-free zone. The general cargo area included 12 warehouses. The grain silo contained 85% of the country's grain, mainly imported from Eastern Europe through vessels and sucked directly into the silos' cells at a 600 mt/h vacuum-suction speed (19) Figure 1. The port channels more than two-thirds of Lebanon's total external trade. With an average annual revenue of USD 313 Million and a net profit of ~USD 124 Million in 2017, the port of Beirut is a vital element of the fragile Lebanese economy, where almost 90% of the port's cargo are imported goods required to satisfy local needs (19).

**Figure 2:**
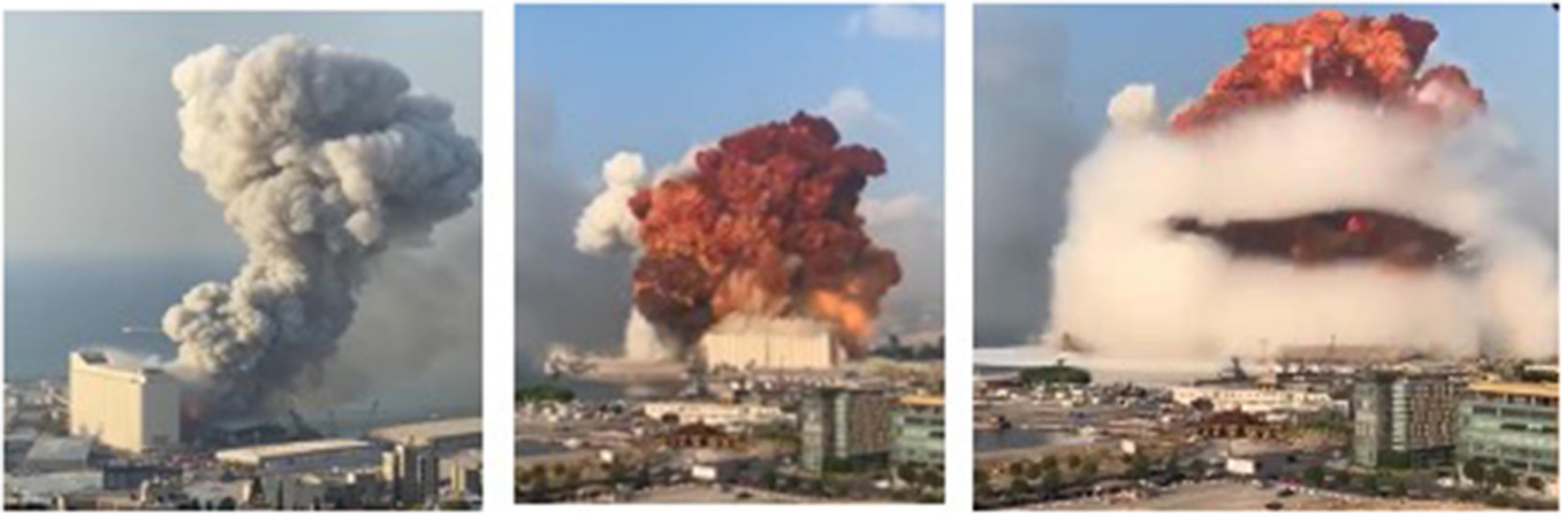
Blast explosion at time 5:55 pm (initial explosion) and at 6:08 pm (orange and mushroom cloud) at the port of Beirut (Source online).

At 5:55 pm local Beirut time, Tuesday August 4, 2020, an uncontrolled fire erupted at a fireworks warehouse in Hangar 12 of the Port of Beirut (PoB). The Beirut fire department deployed a team of nine firefighters and a paramedic to the scene, however, the team failed to control the fierce and intense fire. The warehouse ignited at 6:07 pm causing the first explosion. Approximately 30 s later, a subsequent massive blast occurred in the AN warehouse, next to the grain silos as shown in Figure 2.

**Figure 3:**
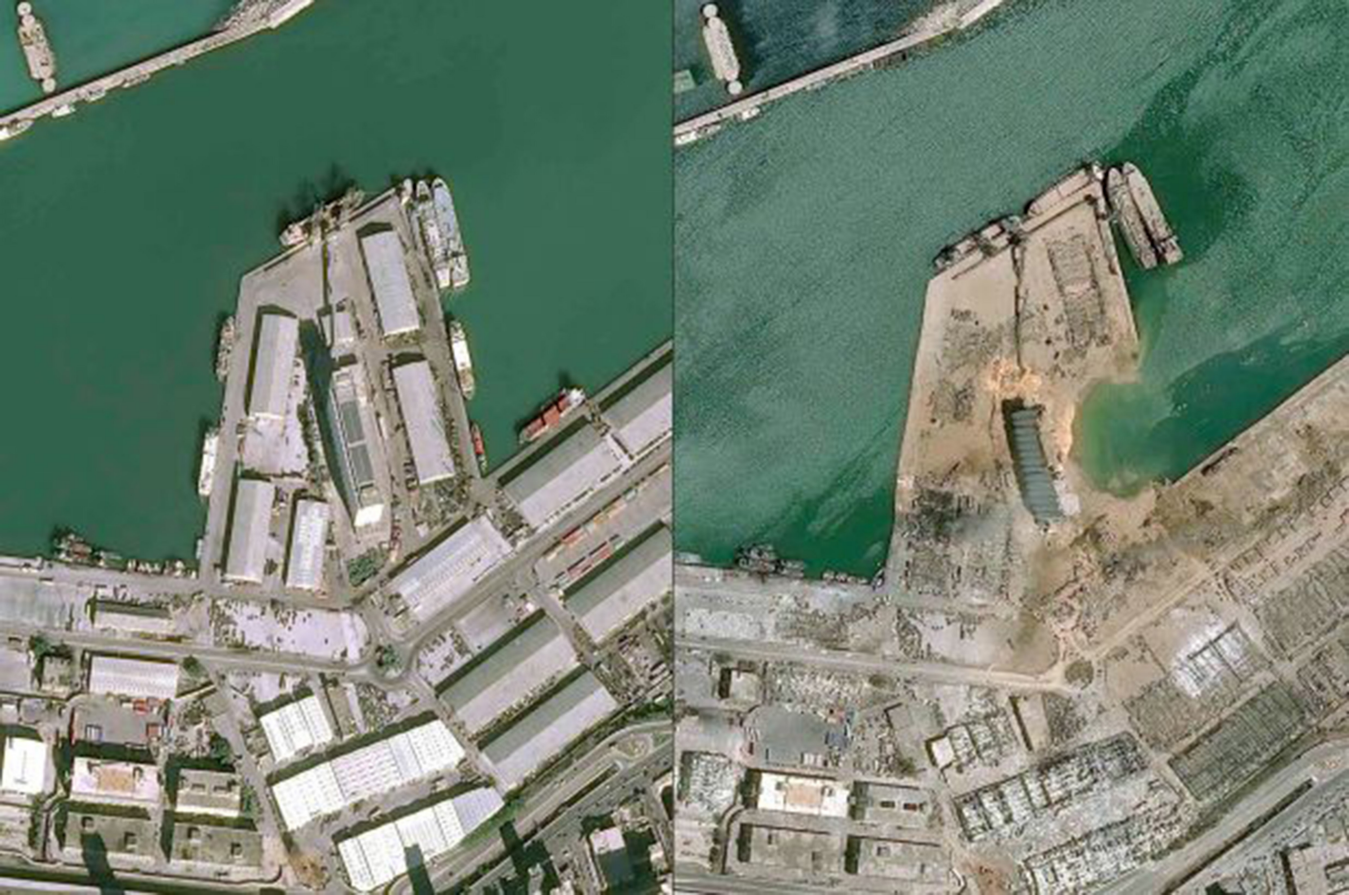
Satellite picture for the Beirut explosion (before and after).

The blast was caused by a large amount of AN exceeding 2.7 kilotons, fueled by the initial flames emitted from the adjacent burning warehouse. The destructive AN detonation resulted in an instantaneous massive blast that was heard in Cyprus 125 miles away in the Mediterranean Sea. The epicenter structures were demolished, along with nearby warehouses, grain silos, and docked ships. The resulting 140-m-wide crater was filled with seawater (Figure 3). In addition to the large ground crater, the explosion created a massive red-orange smoke plume surrounded by a white mushroom pressure cloud. The produced pressure was equivalent to a 3.3 magnitude on a Richter scale, accompanied by a seismic heat and a shockwave that traveled at a supersonic speed, demolishing most of the urban neighborhood around the port and shattering windows as far as 10 km across the Beirut metropolis. Those experiencing the blast felt the earth shake prior to the arrival of the blast wave. In addition to the destruction of most of Beirut port and its grain silos concrete structure, the explosion heavily damaged 50,000 residential houses, along with 178 schools and 9 hospitals, including a children's specialized hospital.

### Health Implications

#### Beirut Blast Injuries

Understanding the common types of injuries associated with blasts is essential to developing an appropriate emergency response and treatment plan. We documented the initial acute management of blast victims presenting to the American University of Beirut Medical Center (AUBMC), a Beirut tertiary hospital of 350-bed capacity, located ~2.5 miles away from the blast site. Injuries were characterized and classified into primary, secondary, tertiary, and quaternary injuries with a particular focus on the neurological manifestations and long-term detrimental outcomes of blast brain injuries.

#### Disaster Acute Management

The unique characteristics of the Beirut disaster imposed a series of challenges to local tertiary hospitals ranging from the provision of acute care and management to the mass casualties to the anticipation of the chronic effects of the blast overpressure and toxic gas exposure. One of these Beirut tertiary hospitals was the American University of Beirut Medical Center (AUBMC). The key challenge that AUBMC faced along with other neighboring hospitals is the urgent need to continue its provision of service and adequate disaster response despite suffering from partial structural damages and sustaining injuries among its hospital staff. Further to dealing with its own emergency situation, the hospital received nearly 360 victims in its 42-capacity Emergency Department (ED). Of those, 270 were treated and released at ED, 108 were admitted and 9 were dead on arrival (20). In the 3 days following the explosion, 68 operations were performed on admitted patients. The greatest diagnostic challenges for the hospital clinicians at all levels of care in the aftermath of the disaster was dealing with the large numbers of casualties and multiple penetrating injuries. Despite activation of the hospital disaster plan, patients presented at a scale much larger than what the hospital resources and capacity could accommodate. Electronic health systems failed to accommodate the surge of patients. The overwhelming surge of injuries hindered formal documentation of patient records; many injured presented without identification. These difficulties to overcome using interim triage and on-site informal record keeping were adopted to achieve effective emergency care (20).

#### Injury Characteristics

**Figure 4:**
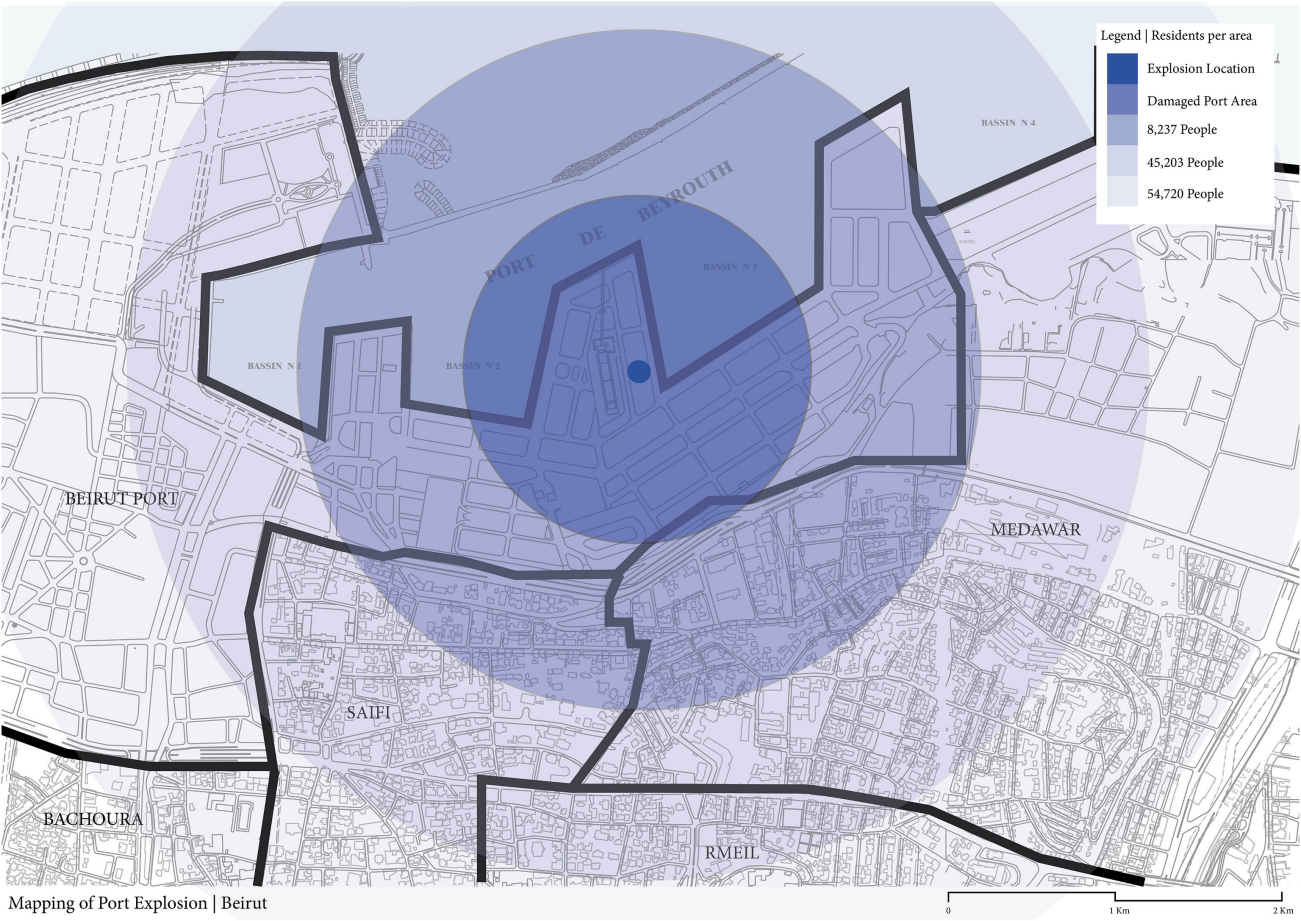
Radius depicting the damages affecting individuals residing at various geographic distances from the blast ranging from 8,237 to 45,203 and reaching out to 54,720 individuals across the Beirut metropolis.

Almost all Port of Beirut employees along with the deployed firefighters at the blast scene died instantly due to their severe injuries. Beirut residents suffered from multiple mechanisms of injuries within a radius of 6 miles from the epicenter and were affected by a mushroom-like cloud of ammonium nitrate (Figure 4). Upon the detonation of Beirut explosive, the initial blast wave produced a millisecond-long supersonic positive pressure gradient wave subsequent to a lengthier negative pressure that expanded outwardly from the blast epicenter across Beirut residential areas (21, 22). Thousands of Beirut disaster victims presented to local hospitals for injury acute management. These victims experienced various mechanisms of blast injuries caused by the explosion pressure and heat wave. The reported injuries align with the Centers for Disease Control and Prevention (CDC) classification of blast injury (23, 24) and were classified into:

##### Primary Injury or Barotrauma

Victims mainly suffered from lung damage and eye/eardrum ruptures due to the blast over-pressurization and under-pressurization waves.

##### Secondary Injury

Victims sustained deep penetrating injuries, traumatic brain injuries and intracranial bleeding, and extremities amputations caused by propelled debris fragments.

##### Tertiary Injury

Victims suffered from ear/eye traumatic injuries, fractured/amputated extremities, and concussion due to structural collapse, and blast wind wave that displaced victims leading to body impact, head acceleration and injury.

##### Quaternary Injury

Victims suffered from toxicity and thermal burns due to gas intoxication and fire. Although a limited number of patients suffered from burns, it is assumed that most burn victims were dead on arrival (DOA).

Some or combinations of these injuring mechanisms were experienced by the blast victims. For further information, please refer to the CDC application (https://apps.apple.com/us/app/cdc-blast-injury/id890434999).

### Environment Implications

#### Beirut Blast Toxicity

The Beirut blast disaster produced enormous white and dark brown fumes covering a large portion of the city. Emission of irritating white fumes and brown fumes characteristically occur during the decomposition of AN. Initially, four chemical species are formed in the gas phase: white ammonia nitrate mist (NH_3_), HNO_3_, nitrous oxide (N_2_O), and H_2_O vapor (25). In confinement, AN completely decomposes allowing for the reaction of the four gases to form water vapor, nitrogen, and toxic brown fumes mainly consisting of nitric oxides (NOx). The most hazardous NOx are nitric oxide (NO) and nitrogen dioxide (NO_2_). Only one or two breaths of the NOx stream can cause severe toxicity. NO_2_ is heavier than air, hence exposure can result in asphyxiation. Odor provides an overt warning for acute exposures. Compared to adults, exposed children may receive greater doses of NO_2_ due to body size and proximity to ground level and the large surface area of their lungs (26).

NO_2_ can damage the respiratory system in several different ways. First, by its conversion into nitric and nitrous acids in the distal airways, damaging alveolar structure. Secondly, by causing oxidative stress through generation of free radicals, which results in protein oxidation, lipid peroxidation, and cell membrane damage (27). In addition, an indirect effect caused by NO_2_ is the alteration of macrophage and immune functions, thereby increasing the risk of lung infections (28). This possibly includes increased risk of COVID-19 infection given that the Beirut blast took place during the ongoing pandemic. A sharp increase in COVID-19 positive cases was observed exactly 10 days after the blast COVID cases increased (from 177 cases on August 3 to 334 cases on August 14) (https://www.moph.gov.lb/en).

The primary site of NOx toxicity usually involves the lower respiratory tract. At low concentrations, symptoms including abdominal pain, nausea, headache, fatigue, coughing, and difficulty breathing are common. In some cases, an asymptomatic period of up to 30 h in exposed subjects may be followed by bronchospasms and pulmonary edema. In cases of an intense exposure, swelling of tissues in the throat and burns may occur, as well as obstruction of the upper respiratory airways (29). The initial effect may also be followed by fibrous obstruction of the bronchioles several weeks later. Such late obstruction presents as a group of additional symptoms including fever and chills, coughing and bleeding in the lungs, cyanosis of the skin, shortness of breath, and in extreme cases a respiratory failure (30). In addition, victims of inhalation may suffer from the reactive airways dysfunction syndrome (RADS), even after an acute exposure. Absorption of NOx into the circulation may lead to methemoglobunemia, a sensation of chest congestion, a dilated heart, and possibly circulatory collapse.

The Beirut explosion also produced large amounts of particulate matter that remained suspended in air for days; this effect has probably impacted more people than any other blast pollutant. Emitted PM may consist of sulfates, nitrates, ammonia, sodium chloride, black carbon, and mineral dust. It includes a complex mixture of organic and inorganic substances suspended in the air. Emitted particles with a diameter of around 10 microns or less (PM_10_) comprise blast demolition dust capable of affecting the upper respiratory airways. Those with a diameter of 2.5 microns or less (PM_2.5_) are released from combustion materials, namely fuel burning. PM_2.5_ may spread into the alveolar region of the lung, and may enter the blood stream, causing a long-term public health concern. Prolonged periods of exposure to respirable particulates PM_2.5_ increases the risk of cardiopulmonary morbidity and mortality (31). Exposed individuals with reactive airways disease may be at higher risk of illness in an environment with elevated PM_10_ and PM_2.5_.

The long-term environmental repercussions of the Beirut explosion remain unexplored. The impact of the AN explosion mushroom cloud smoke, rain precipitation 5 days following the blast, and the fire-water drainage require study. In the current case of the Beirut explosion, chemical release was quite rapid. The resulting toxic plumes were dispersed within 24 h and scattered into the atmosphere to below detectable limits. Given the prevailing wind direction in Lebanon, most of the affected areas were located toward the Northeast, downwind of Ground Zero. In addition, toxic dust was deposited on surfaces and settled on the ground in areas downwind of the port and may have re-suspended in the air with activities during the recovery operations. AN is a plant nutrient, with low toxicity to aquatic life (TLM 96: 10–100 ppm), and is highly biodegradable, hence is not expected to bioconcentrate or accumulate in its original form, particularly that most of it had decomposed in the explosion. On the other hand, the observed rainfall on Day 5 following the blast may have driven re-suspended particulates to runoff into the water supply system and may have dispersed in soil. The impact on drinking water quality and potentially affected soils requires further investigation.

At the same time, environmental and health concerns continue over chemical substances stored at the damaged port warehouses, including 9.5 tons of Class I insecticides which disappeared completely after the blast, along with small amounts of picric acid and methanol, according to the International Environmental Emergency Coordination Cell. In addition, during additional preliminary site surveys conducted one week after the blast, the following substances were found at various warehouses at Port of Beirut: petroleum oils, calcium hypochlorite, benzoyl peroxide, hydrofluoric acids, in addition to other potential hazardous materials, some with insufficient labeling, or are unreachable due to building conditions.

#### Beirut Blast AN Safety Hazard

The Beirut explosion was caused by ~2.75 kiloton of Nitropril™ stored in FIBC bags. Based on the manufacturer's technical data sheet, the product is classified as security sensitive material consisting of a low-density porous white to off-white prilled grade of ammonium nitrate NH_4_NO_3_ (AN), composed of 99% AN, with a total nitrogen mass of 34%. It is specifically designed to be used as an oxidizer in blasting agents. Prilled AN is an acidic water-soluble chemical, with hygroscopic properties. Many studies have reported on the hazardous aspects of products containing AN (32, 33). The related hazards may be classified into three categories: fire, thermal decomposition, and explosion. While AN reacts with organic material, reducing agents, and metal powder, it is not combustible on its own. Nevertheless, its presence increases the intensity of an initiating fire. In cases of a fire where AN is heated above 190–210°C, irreversible decomposition occurs, yielding toxic gases (34).

According to its material safety data sheet (MSDS), NitroprilTM storage requirements include that it be kept dry, away from an ignition or heat source and mainly stored in areas that are well-ventilated. The absence of proper storage conditions coupled with a lengthy storage time (since 2013) in Hangar 12 of the Beirut port, exacerbated the conditions of storage of Nitropril™ bags. The manufacturer warns users that an adjacent detonation or a major fire involves risk of explosion. Confinement of material can also result in detonation according to the product *(write out)* MSDS. Additional dynamics of the Beirut explosion remain uncertain so far. The investigation, however, shows that one or more of these scenarios is likely to have occurred in the context of this explosion. Moreover, in the case of fire, it is recommended by the manufacturer to, first, open up the storage area to provide maximum ventilation, and secondly, to evacuate all personnel to a minimum of 1,000 m away from the site to save first responders and prevent death.

## Discussion

### Characteristics of Existing Ammonium Nitrate Incidents

In the absence of an official report on the root causes of the explosion, we conducted a thorough review of the literature of previous AN incidents to help in gaining insights into the potential root causes of the Beirut disaster. We retrieved information pertaining to AN tragic events that have occurred since 1916. AN blast precipitating factors were investigated including storage techniques (confinement in massive piles), absence of adequate ventilation, chemical contamination (mixture with incompatible materials), humidity (sea air moisture), and exposure to an external thermal source (ignition caused by fire or flames). We further examined AN safety hazard and potential environmental and health implications of these AN incidents compared to the Beirut blast.

Reviewing existing literature, we have identified top AN explosions in each country and compared them to other AN incidents. Since 1916, more than 30 AN tragic events occurred worldwide at industrial sites or during transportation (1, 2, 35–37). We have selected the top AN explosion in each of these countries in terms of its weight and resulting fatalities. Our analysis suggests that multiple interlinked factors typically result in AN detonation (Table 1).

**Table 1:** Characteristics for the top 6 most devastating ammonium nitrate explosions globally.

**Location**	**Year**	**Setting**	**AN in tons**	**Explosion root cause**	**Fatalities**	**Injuries**
Beirut, Lebanon	2020	Port	2,750	Uncontrolled fire ignition	220	6,500
Texas City, USA	1947	Ship	2,086	Uncontrolled fire ignition	581	5,000
Tianjin, China	2015	Port	800	Uncontrolled fire ignition	165	798
Faversham, United Kingdom	1916	AN based factory	700	Uncontrolled fire ignition	115	
Oppau, Germany	1921	AN based factory	450	AN contamination with industrial explosives	561	1,952
New Brunswick, Canada	1947	AN based factory	400	Uncontrolled fire ignition	0	0
Toulouse, France	2001	Plant fertilizer	200	AN contamination with chloride	30	2,242
Tessenderlo, Belguim	1942	AN based factory	150	AN contamination with industrial explosives	189	900
Wayandra, Australia	2014	Transportation	56	Road traffic accident	0	8
Coahuila, Mexico	2007	Transportation	28	Road traffic accident	37	150
Barracas, Spain	2004	Transportation	25	Road Traffic Accident	2	5
Buzau, Romania	2004	Transportation	20	Road traffic accident	18	13
Oulu, Finland	1963	AN based Factory	10	AN contamination with industrial explosives	10	

*See (1, 2, 35, 37–47)*.

Previous AN incidents confirm that uncontrolled fires were the leading root cause for the majority of AN detonation incidents (2, 35, 44, 45). When exposed to heat, high pressure, and temperatures above 190°C, AN decomposes leading to an explosion that may be significantly amplified by confined space storage. Among factors that contributed to the devastating impact of the AN detonation in Beirut is the stockpiling of large amounts of AN in one geographic location which was closely associated with the amplified impact of the explosions in previous similar AN incidents, causing a massive number of casualties (Table 1). Causalities claimed by the AN explosion substantially varied based on the AN storage distance from residential areas. Whether in a factory (37), a fertilizer plant (1, 2) or a warehouse (45), urban AN storage and proximity to residential areas greatly increased the numbers and severity of blast injuries. This proxy effect was clearly demonstrated in the large number of causalities reported at multiple AN incidents in the US, China, and Germany. Our review suggests similarities in lingering factors including violation of safety regulations, coupled with improper AN storage requirements and handling. Secondly, contamination of AN is another root cause that hardens AN stockpiles, exacerbating its hazardous risks and ultimately contributing to a massive detonation as in the case of AN incidents in France and Germany (1, 37).

### Analysis of Environmental and Health Implications of Similar AN Incidents

We have examined the difference and similarities across AN events and in comparison to the Beirut blast. Understanding similar past AN events can provide additional information on blast injuries often classified into four categories based on their mechanisms.

Similar to Beirut blast injury manifestations, brain trauma, pneumothorax, and laceration or contusion of abdominal organs were common primary blast injuries witnessed in the Tianjin and West Texas (46, 47). The Tianjin incident indicates that ears are the most frequently damaged and affected organs in a blast, usually in the form of tympanic membrane perforation (47), while 14% of the 252 injured in the West Texas explosion suffered tinnitus and hearing problems with 5% experiencing tympanic membrane perforation (47).

In the cases of Beirut disaster, residents of nearby populated areas near the port sustained severe injuries from the heatwave and overpressure as blast overpressure was enhanced by the reflections from buildings, blocks, or vehicles. Reflected blast leads to enhanced overpressure effects even after a single blast incident (36). Though not visible, primary blast injury, depending on overpressure, leads to a spectrum of neurological manifestations and brain injuries (48, 49). Mild or moderate blast injuries from primary blast barotrauma often remain uncharacterized and in many cases their chronic effects are undiagnosed as a result of the visible symptoms and the lack of an available specific diagnostic marker (50). Several factors contribute to the extent and pattern of blast injuries including the environment (e.g., surrounding barriers), device composition, and most importantly, the distance between the victim and the blast (50–52).

The difference in the ambient pressure (i.e., the outer atmospheric pressure and the inner pressure) can result in injury of the hollow gas-filled organs [i.e., the lungs, gastrointestinal tract, and the auditory system, traumatic blast brain injury (bTBI)] and in blast TBI and impact /acceleration injuries (21, 23, 52–55). Although blast bTBI may share some characteristics with blunt or even open head TBI, blast-induced non-inertial mTBI is attracting more attention as a unique clinical entity (51, 56, 57).

Similar to Beirut blast, secondary blast injuries were the most observed types of injury (84%) following the Tianjin explosion (47). Blast pressure and wind resulted in the displacement of debris, which caused injury to the head, face, neck, chest, arms, and hands (47). Moreover, several victims of the West Texas incident suffered from lacerations and penetrating trauma, with 12% presenting eye injuries (46). Debris introduced foreign bodies disrupting soft tissues and leading to extensive injury and infection beyond the superficial wound and long-term health complications (58).

As for the tertiary blast injuries, several similarities manifested across AN incidents. The aftermath of the Tianjin explosion led to many traumatic amputations, fractures, concussions, and sprains (46, 47). Similarly, almost 20% of West Texas blast survivors sustained a traumatic head injury or concussion, indicating their high prevalence (46). Post-treatment studies suggest that blast victims are more likely to continue opioid use after discharge and experience reduced improvement in pain intensity (46).

Burns were quaternary blast injuries seen in Tianjin (47). The high prevalence of burn injuries was mainly due to victims' proximity to the center of the incident and their risk of exposure to secondary explosions. Burn injury was associated with greater likelihood of mortality due to their degree of impact on multiple systems (47). Emergency responders often sustain the most cases of burns. Inhalation injuries are also a major source of concern associated with blast incidents, especially due to ammonium nitrate, smoke, and subsequent chemical poisoning (58).

Across all AN incidents, many victims of AN events were reported to suffer from multiple types of blast injuries and their long-term consequences. Furthermore, psychiatric conditions such as post-traumatic stress disorder, anxiety, depression, and substance misuse occur following exposure to an explosion. The medical research community has evaluated in some detail the blast-induced physiologic and pathophysiologic brain alterations due to blast overpressure and impulse (52, 59). The blast induces neuropathological molecular changes in the brain as the blast wave passes through the person's skull and brain water or, in the case of high overpressures which rotate or/and accelerate the head, impact and acceleration injuries occur. Alternatively, indirect hydraulic interactions can be initiated with kinetic energy transfer from the compressed body organs to the body's fluid phase causing oscillating pressure waves, reaching the brain through the venous system (60). Thus, the blast injury mediates its neuropathological effects via different mechanisms. Notably, blast injuries, depending on intensity, may involve macro- and microstructural as well ultrastructural intracellular alterations, changes in cortical thickness and volumetric reduction, and functional network and connectivity changes, as shown in blast mild traumatic brain injury (mild TBI) experimental models (61), as well as diffuse axonal injuries (62–66). Blast injury, mainly mild TBI concussion can result in chronic neurobehavioral and neurostructural change at time points ranging from months to years post-injury (67, 68).

Importantly, the nature of neurobehavioral and neuropathological brain changes attributed to blast mTBI, even with a single blast event, leads to long term detrimental outcomes, due to accumulation of brain-specific proteins such as tau and TDP-43 proteins (54). Afflicted mTBI victims develop later neurobehavioral effects including anxiety, post-traumatic stress disorder (PTSD), depression, and suicidality (67, 69). These individuals may be asymptomatic early but later develop neurocognitive, neuro-psychologic, motor, and consciousness changes at later stages (29, 30). Mild TBI, also referred to as concussion, historically offer considerable challenges not only to the individual but to caregivers and family as well (50, 70). Subsequent to blast injuries, neuropsychological effects of blast and blunt trauma are among the main health concern issues. Many blast victims reported balance problems and as well as neuropsychological impairments such as PTSD, anxiety, and depression. Most of those symptoms described above overlap with those experienced by impact/acceleration injuries that cause mTBI/concussion (71–73). Given the limited success of clinical examination and other diagnostic modalities for assessing TBI generally (74, 75), circulating biomarkers, including UCH-L1, GFAP and most recently NfL, have been found to be sensitive and clinically useful tools to improve the accuracy of diagnosis and outcome prediction, both acutely and in the case of NfL chronically (76–79).

## Actionable Recommendations

Various plausible factors likely triggered the Beirut Blast. Accordingly, lessons learned and a series of actionable recommendations are suggested to promote population safety and improve emergency response.

### Hazard Materials Regulation and Storage Requirement

This explosion marks a serious regulatory failure. The detonation of the AN-based product likely occurred when storage conditions deteriorated: confinement of material, impurity contamination, or a thermal/ignition source. Dangerous reactions between AN and other products include chlorinated compounds, organic materials, and heavy metals, particularly when exposed to the molten AN (1). Investigation of prior AN disasters in different parts of the world guided policy on AN management and safety (Table 1). In Lebanon, the Beirut explosion highlights the need for a national Chemical Regulatory Agency to oversee and implement chemical safety measures and adopt preventive strategies for the entire country. In addition, the local port administration and other concerned authorities require stronger technical support and credentialled inspectors to ensure a high level of safety to improve management of chemical storage facilities, ensure safe storage and handling of chemicals, and develop effective emergency response plans.

An urgent need exists to adopt and enforce standard safety regulations and procedures, particularly in the transport and storage of hazardous materials. The government should introduce safety measures to ensure storage of these materials at suitable distances from population centers and residential areas. Following the West, Texas explosion, President Obama in the United States formed a working group, “Improving Chemical Facility Safety and Security,” that was co-chaired by the Department of Homeland Security, the Environmental Protection Agency (EPA), and the Department of Labor (2). The Lebanese government might consider recruiting stakeholders including practitioners and academicians to identify best practices as part of an executive oversight committee or working group, or as part of the existing Chemical Biological, Radio Nuclear Events Preparedness Program (CBRN). Similar steps would likely improve operational coordination, enhance information sharing between different governmental bodies, and modernize policies and regulations on chemical safety. The suggested committee would coordinate activities across the different governmental entities involved in managing chemicals (e.g., Ministries of Defense, Agriculture, Environment, Public Health…etc.), in order to address safety and security issues as well as to reduce risks associated with hazardous chemicals to handlers, operators, and the community at large. Committee activities may include developing tools, training programs, and resources to strengthen emergency response at the level of the Lebanese Civil Defense, ministries, and municipalities. This could also include sharing information with first responders to build their capacity in planning and responding to similar incidents, reviewing all policies and regulations associated with chemical safety and security, issuing clear guiding documents to educate and raise the awareness of all stakeholders, and coordinating information sharing across the intergovernmental entities communities.

### Laminated Glass in Urban Settings

Given the high risk of secondary blast injuries, the use of laminated glass in urban buildings is critical to reduce building glass fragmentation from explosions and associated consequences. Laminated glass has proven to be effective in combatting blast waves. Following an explosion, glass debris is a known cause of injuries. Laminated glass helps to retain glass fragments on a “polyvinyl butyral (PVB) interlayer upon fracture” (80) while alleviating the effects of scattered and fragmented glass pieces that present a main threat to people following an explosion.

### Hospital Disaster Preparedness

One of the major challenges to medical response was the blast destruction to rural hospitals. Three major hospitals were non-functional, while three others were partially damaged, reducing their ability to admit patients and provide treatment (81). The Beirut explosion produced an overwhelming number of casualties layered upon the escalating national numbers of COVID-19 patients, further straining resources of the healthcare system (82). The massive influx of blast victims exceeded local hospital capacities and the provision of emergency care. Many of Beirut's main hospitals are located near the blast site and were severely damaged, thus reducing hospital capacity in the city by almost one third. Evacuation of damaged hospitals forced the transfer of critically sick COVID-19 patients on ventilators to other health facilities. Many died due to delayed care related to lack of bed capacity. Lessons learned from this tragic disaster should inform the design of hospital preparedness and emergency response plans to deal with other large-scale disasters and mass casualty events.

### Crowd Control and Triaging Away From Hospitals

Crowd control is vital in the aftermath event of an explosion. It must be mediated by responsible parties (i.e., the police) to facilitate the work of first responders. Another major challenge faced by medical responders was the surge of less injured casualties (i.e., the “walking wounded”) first by private transportation or walking to hospitals, while the more injured arrived later by emergency medical services vehicles (83). With limited medical responders and supplies at medical centers, there is a need for crowd control and triaging of simple injuries away from hospitals. Triage mistakes following the Tianjin explosion were major contributors to the surge of medical administration into the hospitals, leading to misallocation of limited resources (47). The walking wounded should be separated from seriously injured casualties through crowd communication and control by a triage officer (83). They can be directed to a collection point to assess their injury severity, determine the degree of urgency and allocate them to a site of treatment accordingly. Establishing first aid sites near the disaster location can reduce crowding of hospitals by less severe cases (83).

### Emergency Response

Adequate preparedness and proper emergency response for chemical spills or chemical-related fire were lacking in Beirut as in many other metropolitan areas. The impact of the explosion was clearly exacerbated by absence of risk reduction and management plans. It is crucial to provide essential training for immediate first responders (firefighters, police, emergency medical services) to adopt safety procedures and avoid toxic exposures. Communication and coordination among multiple entities are key to ensure harmonized multi-site and multi-disciplinary collaboration to provide timely responses. These measures could potentially reduce the adverse impacts of future disasters.

### Investment in Blast Related Health Training

A paucity of blast related training exists in Lebanon, even though the country and the Region is prone to man-made disasters and protracted armed conflicts. In fact, blast injury is the main cause of military TBI during wars in the Middle East, accounting for more than 60% of all combat wounds in current conflicts and the majority of the 423,000 TBI injuries affecting US service members and veterans. This recent Beirut blast events highlights the need to invest in blast related health training for professionals external to military medicine. Training such as STOP THE BLEED (84), is critical to educate health professionals on large-scale disaster preparedness, response, and recovery, in order to save critically injured and to meet similar future disasters.

### Translational Opportunities for Research

This event provides a unique opportunity to assess the chronic effects of blast injury detonation, namely the primary barotrauma due to peak overpressure (within a radius of 6 miles) and the quaternary toxic gases due to the AN intoxication. Apart from the experimental animal studies in blast shock tubes, this unfortunate open blast event provides a translational opportunity to perform a longitudinal study of biomarkers on blast victims that can be tracked across time. Brain-specific mild TBI biomarkers (UCH-L1, NfL, and GFAP) along with inflammatory markers IL-6 and CRP. These measures cans be coupled with a neurological and neuroimaging assessment at chronic time points. It can be anticipated that a proportion of blast injured victims will manifest mental illnesses such as PTSD and depression that need to be recognized and treated a sequalae of brain injury. Diagnostic and treatment measures for such a cohort in Beirut offer the promise of benefit to all those suffering from the disabling effects of traumatic brain injury. This could only be achieved if a detailed registry of all victims is established and maintained, with individual-level information and geocoded data.

## Conclusion

This paper provides an overview of the health and environmental implications of the Beirut explosion. We suggested a series of recommendations as a starting point for health professionals and policymakers to build frameworks, adopt regulations, and implement chemical safety protocols on one hand, and to reshape healthcare system emergency preparedness to be able to respond efficiently to similar large-scale disasters, on the other hand. Future studies should focus on the assessment of physical and emotional blast injuries with a thorough examination of the blast devastating impacts on individuals, communities and the country.

## Author Contributions

SA-H, RD, HD, and FK conceptualized the idea and contributed to drafting the manuscript. SM and HK contributed to manuscript review. All authors have contributed to the editing of the manuscript. All authors have approved the final version of the paper.

## Conflict of Interest

The authors declare that the research was conducted in the absence of any commercial or financial relationships that could be construed as a potential conflict of interest.

## References

[1] Dechy N Bourdeaux T Ayrault N Kordek MA Le Coze JC: First lessons of the Toulouse ammonium nitrate disaster 21st September 2001 AZF plant France. J Hazard Mater. (2004) 111:131–8. 10.1016/j.jhazmat.2004.02.039 15231358

[2] Laboureur DM Han Z Harding BZ Pineda A Pittman WC Rosas C. Case study and lessons learned from the ammonium nitrate explosion at the West Fertilizer facility. J Hazard Mater. (2016) 308:164–72. 10.1016/j.jhazmat.2016.01.039 26812084

[3] Greulich WW Crismon CS Turner ML. The physical growth and development of children who survived the atomic bombing of Hiroshima or Nagasaki. J Pediatr. (1953) 43:121–45. 10.1016/S0022-3476(53)80001-6 13070125

[4] British Broadcasting Corporation (BBC). Beirut Explosion: Lebanon's Government ‘To Resign' As Death Toll Rises (2020). Available online at: https://www.bbc.com/news/world-middle-east-53720383 (accessed January 28, 2021).

[5] World Health Organization (WHO). Beirut Port Blast Emergency Strategic Response Plan (2020). Available online at: http://www.emro.who.int/images/stories/lebanon/who-lebanon-strategic-response-plan-27.9.20.pdf?ua=1 (accessed January 28, 2021).

[6] The World Bank. Beirut Rapid Damage and Needs Assessment (RDNA) (2020). Available online at: https://www.worldbank.org/en/country/lebanon/publication/beirut-rapid-damage-and-needs-assessment-rdna—august-2020 (accessed August 2020).

[7] Abdul-Nabi SS Sawaya RD. Airway breathing circulation: an emergency medicine resident's experience of the Beirut explosion. Acad Emerg Med. (2020) 28:483–6. 10.1111/acem.14147 33022837

[8] Al-Hajj S Mokdad AH Kazzi A. Beirut explosion aftermath: lessons and guidelines. Emerg Med J. (2021). 10.1136/emermed-2020-210880. [Epub ahead of print]. 33687991 PMC11524527

[9] Cheaito MA Al-Hajj S. A brief report on the Beirut port explosion. Mediterr J Emerg Med Acute Care. (2020) 9. Available online at: https://excholarship.org/uc/item/6zn9zlj9

[10] Guglielmi G. Why Beirut's ammonium nitrate blast was so devastating. Nature. (2020). 10.1038/d41586-020-02361-x 32778708

[11] Hagon O Dumont L. The Beirut blast 2020: lessons learned from the Swiss emergency medical team specialized “mother & child. *Am J Disaster Med*. (2020) 15:303–5. 10.5055/ajdm.2020.0379 33428201

[12] Landry MD Alameddine M Jesus TS Sassine S Koueik E Raman SR. The 2020 blast in the Port of Beirut: can the Lebanese health system” build back better"? BMC Health Serv Res. (2020) 20:1040. 10.1186/s12913-020-05906-y 33183285 PMC7659403

[13] Mansour H Bitar E Fares Y Makdessi A Maalouf A El Ghoul M. Beirut Port Ammonium Nitrate Explosion.

[14] Pasman HJ Fouchier C Park S Quddus N Laboureur D. Beirut ammonium nitrate explosion: are not we really learning anything? Process Saf Prog. (2020) 39:e12203. 10.1002/prs.12203

[15] Sivaraman S Varadharajan S. Investigative consequence analysis: a case study research of Beirut explosion accident. J Loss Prev Process Ind. 69:104387. 10.1016/j.jlp.2020.104387

[16] Stennett C Gaulter S Akhavan J. An estimate of the TNT-equivalent net explosive quantity (NEQ) of the Beirut port explosion using publicly-available tools and data. Propellants Explosives Pyrotechnics. (2020) 45:1675–9. 10.1002/prep.202000227

[17] Ur Rehman S Ahmed R Ma K Xu S Aslam MA Bi H. Ammonium nitrate is a risk for environment: a case study of Beirut (Lebanon) chemical explosion and the effects on environment. Ecotoxicol Environ Saf . (2021) 210:111834. 10.1016/j.ecoenv.2020.111834 33401200

[18] Zaiter M Ayoub A Mohana A Guermazi A Jarraya M. Beirut port explosion: unusual presentation of bilateral blast-related extensor mechanism rupture. Skeletal Radiol. (2021) 50:1–5. 10.1007/s00256-020-03707-2 33506315

[19] Logistics Capacity Assessments (LCAs). Atlassian by the World Food Program (2020). Available online at: https://dlca.logcluster.org/display/public/DLCA/2.1.1+Lebanon+Port+of+Beirut (accessed January 22, 2021).

[20] The American College of Surgeons. Mass Casualty Trauma Management. Early Lessons from Beirut (2020). Available online at: https://www.facs.org/international/webinar/mass-casualty-management (accessed January 11, 2021).

[21] DeWitt DS Prough DS. Blast-induced brain injury and posttraumatic hypotension and hypoxemia. J. Neurotrauma. (2009) 26:877–87. 10.1089/neu.2007.0439 18447627

[22] Bryden DW Tilghman JI Hinds SR. Blast-related traumatic brain injury: current concepts and research considerations. J Exp Neurosci. (2019) 13:1179069519872213. 10.1177/1179069519872213 31548796 PMC6743194

[23] DePalma RG Burris DG Champion HR Hodgson MJ. Blast injuries. N Engl J Med. (2005) 352:1335–42. 10.1056/NEJMra042083 15800229

[24] Centers for Disease Control and Prevention (CDC). Explosions and Blast Injuries A Primer for Clinicians. (2003). Available online at: https://www.cdc.gov/masstrauma/preparedness/primer.pdf (accessed March 2021).

[25] Gunawan R Zhang D. Thermal stability and kinetics of decomposition of ammonium nitrate in the presence of pyrite. J Hazard Mater. (2009) 165: 751–8. 10.1016/j.jhazmat.2008.10.054 19056177

[26] Centers for Disease Control and Prevention (CDC). Nitrogen Oxides (NO, NO_2_, and others) (2020). Available online at: https://wwwn.cdc.gov/TSP/MMG/MMGDetails.aspx?mmgid=394&toxid=69 (accessed January 23, 2021).

[27] Kagawa J. Evaluation of biological significance of nitrogen oxides exposure. Tokai J Exp Clin Med. (1985) 10:348–53. 3836516

[28] Chauhan AJ Krishna MT Frew AJ Holgate ST. Exposure to nitrogen dioxide (NO_2_) and respiratory disease risk. Rev Environ Health. (1998) 13:73-90. 9718623

[29] Kampa M Castanas E. Human health effects of air pollution. Environ Pollut. (2008) 151:362–7. 10.1016/j.envpol.2007.06.012 17646040

[30] Wegmann M Fehrenbach A Heimann S Fehrenbach H Renz H Garn H. NO_2_-induced airway inflammation is associated with progressive airflow limitation and development of emphysema-like lesions in C57BL/6 mice. Exp Toxicol Pathol. (2005) 56:341–50. 10.1016/j.etp.2004.12.004 15945273

[31] Hoek G Krishnan RM Beelen R Peters A Ostro B Brunekreef B. Long-term air pollution exposure and cardio- respiratory mortality: a review. Environ Health. (2013) 12:43. 10.1186/1476-069X-12-43 23714370 PMC3679821

[32] United States Environmental Protection Agency (USPE). Chemical Safety Alert: Explosion Hazard From Ammonium Nitrate, EPA 550-F-97-002d. (1997). Available online at: https://archive.epa.gov/emergencies/docs/chem/web/pdf/ammonitr.pdf (accessed Jan 15 2021).

[33] Van Dolah R Mason C Perzak F Hay J Forshey D. Explosion Hazards of Ammonium Nitrate Under Fire Exposure. U. S. Dept. of the Interior, Bureau of Mines (1966). Available online at: https://www.osmre.gov/resources/blasting/docs/USBM/RI6773ExplosionHazardsAmmoniumNitrateUnderFireExposure.pdf (accessed March, 2021).

[34] Marlair G Kordek MA. Safety and security issues relating to low capacity storage of AN-based fertilizers. J Hazard Mater. (2005) 123:13–28. 10.1016/j.jhazmat.2005.03.028 15885898

[35] Stephens H. The Texas City Disaster. Austin, TX: University of Texas Press (1997).

[36] Watts S Kirkman E Bieler D Bjarnason S Franke A Gupta R. Guidelines for using animal models in blast injury research. J R Army Med Corps. (2019) 165:38–40. 10.1136/jramc-2018-000956 29643122

[37] Robertson R Thomas HH Hallimond AF Bragg WRT. Investigation on the chemical and physical properties of Oppau ammonium sulphate-nitrate at the government laboratory. Trans Faraday Soc. (1924) 20:46–55. 10.1039/tf9242000046

[38] Kaleva. Finland Explosion (1963). Available online at: https://www.kaleva.fi/vuonna-1963-typpi-rajahti-lahtemattomasti-oululais/2240951 (accessed March, 2021).

[39] Wikipedia. Mihailsti Explosion (2004). https://en.wikipedia.org/wiki/Mih%C4%83ile%C8%99ti_explosion (accessed March 2021).

[40] Atfield C. Truck Explosion Injures Eight, Closes Mitchell Highway (2014). Available online at: https://www.nytimes.com/2007/09/10/world/americas/10cnd-mexico.html (accessed March 2021).

[41] Tessenderlo Group. Explosion at the Produits Chimiques de Tessenderloo (PCT) Factory, Wednesday 29 April 1942 (1942). Available online at: https://www.100yearstessenderlo.com/en/home/1942/april-29-1942-explosion (accessed March 2021).

[42] McKinley J. Truck Explosion in Mexico Kills 37 (2007). Available online at: https://www.nytimes.com/2007/09/10/world/americas/10cnd-mexico.html (accessed March 2021).

[43] Faversham Times. Tales of Horror and Heroism After the Great Explosion (2012). Available online at: https://web.archive.org/web/20140409013355/http://www.canterburytimes.co.uk/Tales-horror-heroism-Great-Explosion/story-15667862-detail/story.html (accessed March 2021).

[44] BBC. Beirut Explosion: Lebanon's Government 'to Resign' as Death Toll Rises (2020). Available online at: https://www.bbc.com/news/world-middle-east-53720383 (accessed September 21, 2020).

[45] Zhao B. Facts and lessons related to the explosion accident in Tianjin Port, China. Nat Hazards. (2016) 84:707–13. 10.1007/s11069-016-2403-0

[46] Metzger K Akram H Feldt B Stone K Alvey S Henley S. Epidemiologic investigation of injuries associated with the 2013 fertilizer plant explosion in West, Texas. Disaster Med Public Health Preparedness. (2016) 10:583–90. 10.1017/dmp.2015.186 26932770

[47] Yu M Lv Q Ding H Zeng X Cao J Liu J. Evaluation of blast injury patients from the 2015 Tianjin explosions in China. Burns. (2016) 42:1133–40. 10.1016/j.burns.2016.03.004 27311537

[48] Rafaels KA Bass CR Panzer MB Salzar RS Woods WA Feldman SH. Brain injury risk from primary blast. J Trauma Acute Care Surg. (2012) 73:895–901. 10.1097/TA.0b013e31825a760e 22836001

[49] Song H Cui J Simonyi A Johnson CE Hubler GK DePalma RG. Linking blast physics to biological outcomes in mild traumatic brain injury: Narrative review and preliminary report of an open-field blast model. Behav Brain Res. (2018) 340:147–58. 10.1016/j.bbr.2016.08.037 27555538

[50] Belanger HG Scott SG Scholten J Curtiss G Vanderploeg RD. Utility of mechanism-of-injury-based assessment and treatment: blast injury program case illustration. J Rehabil Res Dev. (2005) 42:403–12. 10.1682/JRRD.2004.08.0095 16320137

[51] Mayorga MA. The pathology of primary blast overpressure injury. Toxicology. (1997) 121:17–28. 10.1016/S0300-483X(97)03652-4 9217312

[52] de Lanerolle NC Hamid H Kulas J Pan JW Czlapinski R Rinaldi A. Concussive brain injury from explosive blast. Ann Clin Transl Neurol. (2014) 1:692–702. 10.1002/acn3.98 25493283 PMC4241796

[53] Cernak I. Understanding blast-induced neurotrauma: how far have we come? Concussion. (2017) 2:CNC42. 10.2217/cnc-2017-0006 30202583 PMC6093818

[54] DePalma RG Hoffman SW. Combat blast related traumatic brain injury (TBI): decade of recognition; promise of progress. Behav Brain Res. (2018) 340:102–5. 10.1016/j.bbr.2016.08.036 27555540

[55] DePalma RG. Combat TBI: history, epidemiology, and injury modes. In: Kobeissy FH editor. Brain Neurotrauma: Molecular, Neuropsychological, and Rehabilitation Aspects. Boca Raton, FL: CRC Press (2015). 10.1201/b18126-3

[56] Ling G Bandak F Armonda R Grant G Ecklund J. Explosive blast neurotrauma. J Neurotrauma. (2009) 26:815–25. 10.1089/neu.2007.0484 19397423

[57] de Lanerolle NC Bandak F Kang D Li AY Du F Swauger P. Characteristics of an explosive blast-induced brain injury in an experimental model. J Neuropathol Exp Neurol. (2011) 70:1046–57. 10.1097/NEN.0b013e318235bef2 22002430

[58] Finlay SE Earby M Baker DJ Murray VS. Explosions and human health: the long-term effects of blast injury. Prehospital Disaster Med. (2012) 27:385. 10.1017/S1049023X12000891 22800859

[59] Duckworth JL Grimes J Ling GS. Pathophysiology of battlefield associated traumatic brain injury. Pathophysiology. (2013) 20:23–30. 10.1016/j.pathophys.2012.03.001 22703708

[60] Cernak I Wang Z Jiang J Bian X Savic J. Ultrastructural and functional characteristics of blast injury-induced neurotrauma. J Trauma. (2001) 50:695–706. 10.1097/00005373-200104000-00017 11303167

[61] Song H Konan LM Cui J Johnson CE Hubler GK DePalma RG. Nanometer ultrastructural brain damage following low intensity primary blast wave exposure. Neural Regen Res. (2018) 13:1516–9. 10.4103/1673-5374.237110 30127104 PMC6126131

[62] Davenport ND Lim KO Armstrong MT Sponheim SR. Diffuse and spatially variable white matter disruptions are associated with blast-related mild traumatic brain injury. Neuroimage. (2012) 59:2017–24. 10.1016/j.neuroimage.2011.10.050 22040736

[63] Kasahara K Hashimoto K Abo M Senoo A. Voxel- and atlas-based analysis of diffusion tensor imaging may reveal focal axonal injuries in mild traumatic brain injury – comparison with diffuse axonal injury. Magn Reson Imaging. (2012) 30:496–505. 10.1016/j.mri.2011.12.018 22285880

[64] Niogi SN Mukherjee P: Diffusion tensor imaging of mild traumatic brain injury. J Head Trauma Rehabil. (2010) 25:241–55. 10.1097/HTR.0b013e3181e52c2a 20611043

[65] Niogi SN Mukherjee P Ghajar J Johnson C Kolster RA Sarkar R. Extent of microstructural white matter injury in postconcussive syndrome correlates with impaired cognitive reaction time: a 3T diffusion tensor imaging study of mild traumatic brain injury. AJNR Am J Neuroradiol. (2008) 29:967–73. 10.3174/ajnr.A0970 18272556 PMC8128563

[66] Mac Donald CL Johnson AM Cooper D Nelson EC Werner NJ Shimony JS. Detection of blast-related traumatic brain injury in U.S. military personnel. N Engl J Med. (2011) 364:2091–100. 10.1056/NEJMoa1008069 21631321 PMC3146351

[67] >Warden D: Military TBI during the Iraq and Afghanistan wars. J Head Trauma Rehabil. (2006) 21:398–402. 10.1097/00001199-200609000-00004 16983225

[68] Phipps H Mondello S Wilson A Dittmer T Rohde N Schroeder P. Characteristics impact of U.S. military blast-related mild traumatic brain injury: a systematic review. Front. Neurol. (2020) 11:559318. 10.3389/fneur.2020.559318 33224086 PMC7667277

[69] Chen Y Huang W. Non-impact, blast-induced mild TBI and PTSD: concepts and caveats. Brain Inj. (2011) 25:641–50. 10.3109/02699052.2011.580313 21604927

[70] Lew HL. Rehabilitation needs of an increasing population of patients: Traumatic brain injury, polytrauma, and blast-related injuries. J Rehabil Res Dev. (2005) 42:13–6. 10.1682/JRRD.2005.07.0124 16320135

[71] King NS. PTSD and traumatic brain injury: folklore and fact? Brain Inj. (2008) 22:1–5. 10.1080/02699050701829696 18183503

[72] Lew HL Vanderploeg RD Moore DF Schwab K Friedman L Yesavage J. Overlap of mild TBI and mental health conditions in returning OIF/OEF service members and veterans. J Rehabil Res Dev. (2008) 45:11–6. 10.1682/JRRD.2008.05.0064 18629743

[73] Kennedy JE Leal FO Lewis JD Cullen MA Amador RR. Posttraumatic stress symptoms in OIF/OEF service members with blast-related non-blast-related mild TBI. NeuroRehabilitation. (2010) 26:223–31. 10.3233/NRE-2010-0558 20448312

[74] Mondello S Muller U Jeromin A Streeter J Hayes RL Wang KK. Blood-based diagnostics of traumatic brain injuries. Expert Rev Mol Diagn. (2011) 11:65–78. 10.1586/erm.10.104 21171922 PMC3063529

[75] Mondello S Schmid K Berger RP Kobeissy F Italiano D Jeromin A. The challenge of mild traumatic brain injury: role of biochemical markers in diagnosis of brain damage. Med Res Rev. (2014) 34:503–31. 10.1002/med.21295 23813922

[76] Carr W Yarnell AM Ong R Walilko T Kamimori GH da Silva U. Ubiquitin carboxy-terminal hydrolase-l1 as a serum neurotrauma biomarker for exposure to occupational low-level blast. Front Neurol. (2015) 6:49. 10.3389/fneur.2015.00049 25852633 PMC4360700

[77] Peltz CB Kenney K Gill J Diaz-Arrastia R Gardner RC Yaffe K. Blood biomarkers of traumatic brain injury and cognitive impairment in older veterans. Neurology. (2020) 95:e1126–33. 10.1212/WNL.0000000000010087 32571850 PMC7538225

[78] Gill J Latour L Diaz-Arrastia R Motamedi V Turtzo C Shahim P. Glial fibrillary acidic protein elevations relate to neuroimaging abnormalities after mild TBI. Neurology. (2018) 91:e1385–9. 10.1212/WNL.0000000000006321 30209234 PMC6177279

[79] Mondello S Sorinola A Czeiter E Vamos Z Amrein K Synnot A. Blood-based protein biomarkers for the management of traumatic brain injuries in adults presenting to emergency departments with mild brain injury: a living systematic review and meta-analysis. J Neurotrauma. (2018) 38:1086–106. 10.1089/neu.2017.5182 29020853 PMC8054517

[80] Hooper P Sukhram R Blackman B Dear J. On the blast resistance of laminated glass. Int J Solids Struct. (2012) 49:899–918. 10.1016/j.ijsolstr.2011.12.008

[81] World Bank. Beirut Rapid Damage and Needs Assessment (2020). Available online at: https://www.worldbank.org/en/country/lebanon/publication/beirut-rapid-damage-and-needs-assessment-rdna—august-2020 (accessed March 2021).

[82] Al-Hajj S Abou-El-Hassan H Khalil L Kaafarani H El Sayed M. Hospital disaster and emergency preparedness (HDEP) in Lebanon: a national comprehensive assessment. Int J Disaster Risk Reduct. (2020) 51:101889. 10.1016/j.ijdrr.2020.101889

[83] El Sayed MJ. Beirut ammonium nitrate explosion: a man-made disaster in times of CoViD19 pandemic. Disaster Med Public Health Preparedness. (2020):1–18. 10.1017/dmp.2020.451. [Epub ahead of print]. PMC7985624 33203497

[84] Stopthebleed. Stopthebleed (2020). Available online at: https://www.stopthebleed.org (accessed March 2021).

